# Validation of deep learning-based markerless 3D pose estimation

**DOI:** 10.1371/journal.pone.0276258

**Published:** 2022-10-20

**Authors:** Veronika Kosourikhina, Diarmuid Kavanagh, Michael J. Richardson, David M. Kaplan

**Affiliations:** 1 School of Psychological Sciences, Faculty of Medicine, Health and Human Sciences, Macquarie University, Sydney, Australia; 2 The MARCS Institute for Brain, Behaviour and Development, Western Sydney University, Parramatta, Australia; 3 International Centre for Neuromorphic Systems, Western Sydney University, Parramatta, Australia; 4 Centre for Elite Performance, Expertise and Training, Macquarie University, Sydney, Australia; 5 Perception in Action Research Centre, Macquarie University, Sydney, Australia; Linköping University: Linkopings universitet, SWEDEN

## Abstract

Deep learning-based approaches to markerless 3D pose estimation are being adopted by researchers in psychology and neuroscience at an unprecedented rate. Yet many of these tools remain unvalidated. Here, we report on the validation of one increasingly popular tool (DeepLabCut) against simultaneous measurements obtained from a reference measurement system (Fastrak) with well-known performance characteristics. Our results confirm close (mm range) agreement between the two, indicating that under specific circumstances deep learning-based approaches can match more traditional motion tracking methods. Although more work needs to be done to determine their specific performance characteristics and limitations, this study should help build confidence within the research community using these new tools.

## Introduction

Recent advances in computer vision and machine learning have catalyzed the development of powerful tools for markerless 3D pose estimation [1, 2]. Although these tools afford promising new opportunities for rapid, efficient quantitative measurement of animal and human behavior in psychology, neuroscience, and a range of other fields [3], their accuracy and reliability have not been rigorously established. This situation puts the user community at risk and places scientific results that depend on these tools on uncertain foundations. Although less frequently acknowledged, measurement differences are another potential factor underlying the failure of reproducibility of experimental results across studies which has led to a recent crisis of confidence in psychology and neuroscience [4, 5]. In these fields, where the employment of a diverse range of methods for measuring behavior (and brain activity) is commonplace, confirming that different methods produce consistent, comparable values is critical for scientific progress.

Here, we describe the validation of one increasingly popular open-source deep learning-based pose estimation tool–DeepLabCut (DLC v2.1.3; [6, 7]). We systematically compared 3D kinematic data generated via a DLC-based workflow using video frames collected from two cameras positioned at different viewing angles against simultaneous measurements obtained from a reference measurement system with well-known performance characteristics. We selected Fastrak (Polhemus, Vermont, USA), an electromagnetic 3D motion tracking system, as our reference system because of its high accuracy (static position accuracy: 0.76 mm RMS) and its wide use across human neuroscience and psychology.

## Materials and methods

### Experimental setup

**Fig 1:**
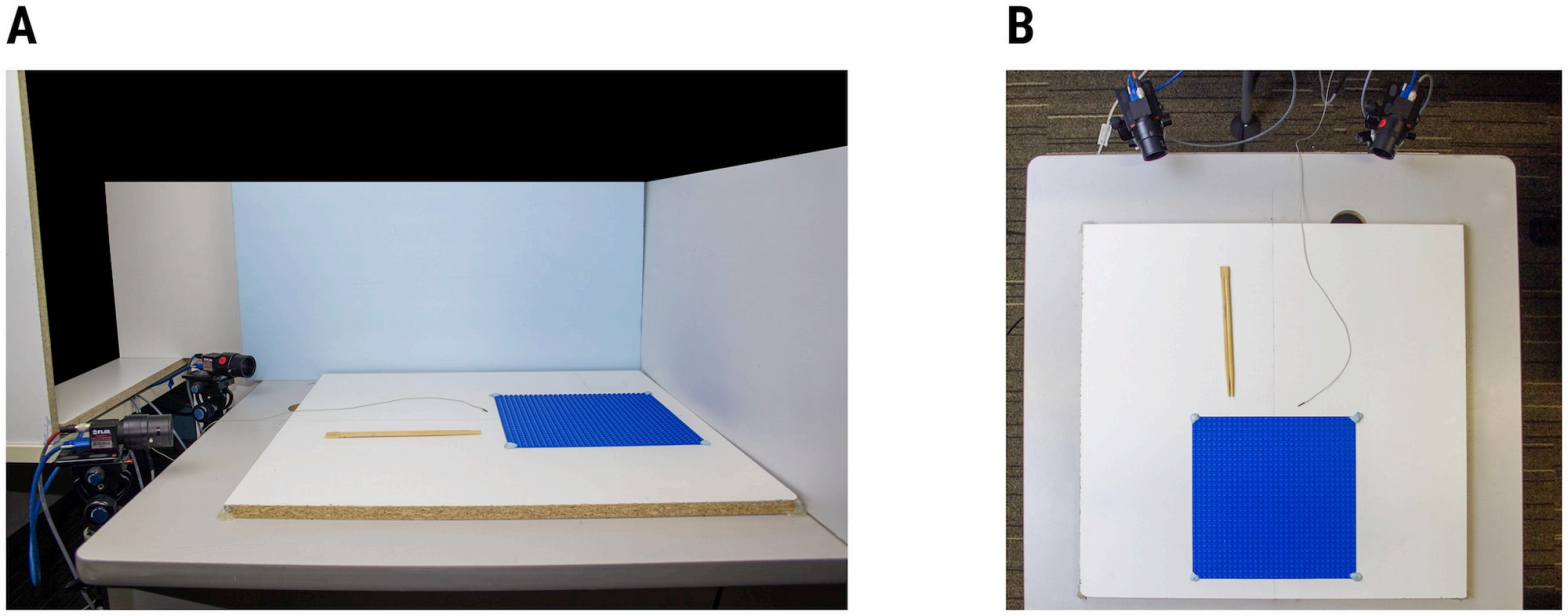
DLC setup as seen from (a) side and (b) overhead views. The Lego plate marks the boundaries of the testing area. Wooden dowels and the Fastrak microsensor are shown separately for easier visualization.

Data was collected with Polhemus Fastrak (Micro Sensor 1.8) and two cameras (Blackfly S BFS-U3-04S2M). The filming volume was centered on a Lego plate securely attached to a non-ferrous table (Fig 1). A Fastrak transmitter was attached to the underside of the table, such that the entire filming volume remained within one hemisphere of the transmitter (Y+). The cameras were placed on stands attached to a platform at one side of the filming table. The inner edges of the stands were 30cm apart and camera lenses were positioned approximately 5cm above the table surface. The center of the filming volume was approximately 60cm from the outer surface of the camera lenses. A laser pointer was attached on top of each camera to verify that the cameras were pointed at the same central point a few centimeters above the filming volume during all recordings. Note that cameras had to be positioned with minimal pitch relative to the filming volume, because the calibration process does not handle camera pitch well and produces 3D coordinates that appear to be tilted towards the cameras (i.e., Z-axis coordinates increased disproportionately further away from the cameras).

To obtain concurrent data, we used DLC to track the position of a Fastrak microsensor. In the tool version of the tasks, the sensor was attached to the ridge formed between two joined dowels. To improve the accuracy and precision of DLC labelling, crosshairs were drawn over the center of the microsensor. In the finger version of the tasks, the microsensor was attached at the center of the fingernail of the left index finger. Both DLC and Fastrak were set up to have the same origin point (one corner of a Lego brick located in the center of the filming volume). Both systems measured the position of the sensor relative to the point of origin in three dimensions (in camera view, X-axis was left-right, Y-axis was depth, and Z-axis was up-down). Euclidean distance was calculated as a summary metric.

### Frame synchronization

Obtaining accurate 3D data and accurate comparisons with Fastrak data requires the video recordings to be synchronized across the two cameras and with Fastrak. Synchronization between the two cameras and Fastrak was achieved as follows. Camera 1 was controlled directly from the computer, and Camera 2 and Fastrak were set up to start and stop recording on signal from Camera 1. Cameras 1 and 2 were connected via a GPIO synchronization cable to a digital I/O device (NI USB-6501), which was in turn connected to the computer. Fastrak was also connected to both the computer and the I/O device in order to detect the signal that Camera 1 sent to trigger Camera 2 to start and stop recording.

All three devices recorded with the same frequency (120 frames per second for the cameras, 120Hz for Fastrak), allowing for maximum temporal synchronization between data points from Fastrak and DLC. In some cases, there was a small lag in stopping the Fastrak recording (~8 data points)–only the first 1200 or 2400 points (depending on task) were analyzed. To make the data directly comparable, we set Fastrak’s reference point to match the origin point used for camera calibration (see Calibration section). Fastrak’s settings and recording were controlled with a Matlab script, and the cameras were controlled with Multi-Pyspin (https://github.com/justinblaber/multi_pyspin). Multi-Pyspin’s code was edited to collect diagnostic information and to optimize the frame acquisition process and improve synchronization.

### Camera calibration

Accurate 3D pose estimation requires precise camera calibration, which establishes the relative location and various parameters of each camera such as the focal length and lens distortion. We chose to use the easyWand software tool [8], over some other available alternatives including DLC’s built-in camera calibration tool (3D DLC) and Anipose [9] as we were not able to produce reliable calibrations with these tools. The decision to use easyWand is by no means a disparagement of other available camera calibration tools. Additionally, both 3D DLC and Anipose have likely undergone further development since our early piloting and may no longer have the issues we experienced when using these tools. During our initial attempts, we experienced several undocumented issues including the fact that idiosyncrasies in the filmed videos seemed to change calibration quality or crash the process entirely. New users should anticipate potential difficulties in obtaining quality calibrations and should allocate some time to piloting their setup with their chosen software. In some cases, using tools that directly integrate with DLC (such as 3D DLC or Anipose) could be sufficient and more practical.

We settled on using easyWand because we found it to be the most controllable and reliable of the three calibration tools we considered. While the other tools use checker or ChArUco boards for calibration videos and can automatically find 2D coordinates of points needed for calibration, we found this step to be unreliable. EasyWand requires manual labelling of the necessary points (2 wand points and 4 axis points, see below for details). Although more labor-intensive, we found this process to work more reliably–almost every calibration video we filmed produced a good calibration. EasyWand also allowed us to easily align the reference frame of the calibration to Fastrak’s reference frame, which simplified subsequent analyses. We used easyWand to calculate direct linear transformation coefficients and convert 2D pixel coordinates into real 3D spatial coordinates. We filmed a set of calibration videos with the necessary points labelled in DLC and used the pair with the lowest reprojection error for calculating the 3D coordinates used in the current analyses.

To calculate direct linear transformation (DLT) coefficients, easyWand requires a dataset with 2D coordinates for a set of two "wand points” and four "axis points” from each camera. Wand points are two points positioned at a fixed distance from each other that are moved throughout the volume of interest. We used the same joined wooden dowels as in the tool task, using the crosshairs over the Fastrak sensor as one point, and another set of crosshairs marked 10cm away as the second point. The axis points (one origin point and three points for each of the axes) allow aligning of the data to a particular set of axes. To get precise axis points, we placed a Lego cube and an additional Lego brick in the center of the filming volume and labelled three corners of the cube and one corner of the separate brick as the axis points. All three axis points were 31mm away from the origin point.

We filmed three pairs of calibration videos (20 seconds each), making waving motions with the dowel throughout the volume of interest. After obtaining 2D coordinates for the wand and axis points with DLC, we replaced points with < .95 likelihood with NaNs and inverted Y-axis values (by subtracting raw coordinates from image height in pixels). The latter was done to change coordinates into a bottom-oriented system, to match that apparently used by easyWand. Using raw values has consistently produced nonsense results. We saved every 10th datapoint for the list of wand-point coordinates to use in the calibration (producing ~120–150 usable points), because using the entire set (2400 points) made it more difficult to get accurate calibrations. For axis points, easyWand only takes one coordinate point for each, so we calculated median values from our full dataset to get Origin, X, Y, and Z-axis reference point coordinates.

Intrinsic camera parameters were included to improve calibration quality: image height (540px), image width (720px), focal length (1450px), and principal point (360px). For the calibration used to acquire current data, we set the calibration to use focal length only, and applying all lens distortion coefficients. We also excluded outliers until a reprojection error of .28 was achieved with sensible estimates of camera positions. Original reprojection error (.54) was not overly large, but piloting suggested that reprojection errors < .30 are needed to achieve this degree of agreement with our setup.

### Data processing

After all data was collected, we checked timestamps from both videos and Fastrak to confirm that the correct number of data points were recorded at approximately equal time intervals. No issues were observed. To train a DLC network, we labelled 20 frames from each of two videos (camera 1 and 2) from one randomly selected trial of each task (center-out tool: trial 3; zigzag tool: trial 11; center-out finger: trial 11; zigzag finger: trial 6). Frame labelling and network training was done with default DLC settings, following the published protocol (Nath et al., 2019).

To convert 2D pixel coordinates into 3D spatial coordinates, we processed our data with a Matlab script adapted from another markerless tracking software that accepts calibration coefficients in easyWand’s format (DLTdv7, [10]; https://github.com/tlhedrick/dltdv). After aligning 3D data with Fastrak, we excluded any points that had < .95 likelihood in the original 2D pixel format. Plotting the remaining data as 3D scatterplots showed an apparent glitch in the Fastrak data for zigzag tasks, where its trajectory clearly deviated from the real motion path (and therefore DLC data) in one small part of the filming volume. This segment of the data (X ±40mm, Y < -120mm, and Z < 25mm) was excluded from further analyses.

### Experimental tasks

**Fig 2:**
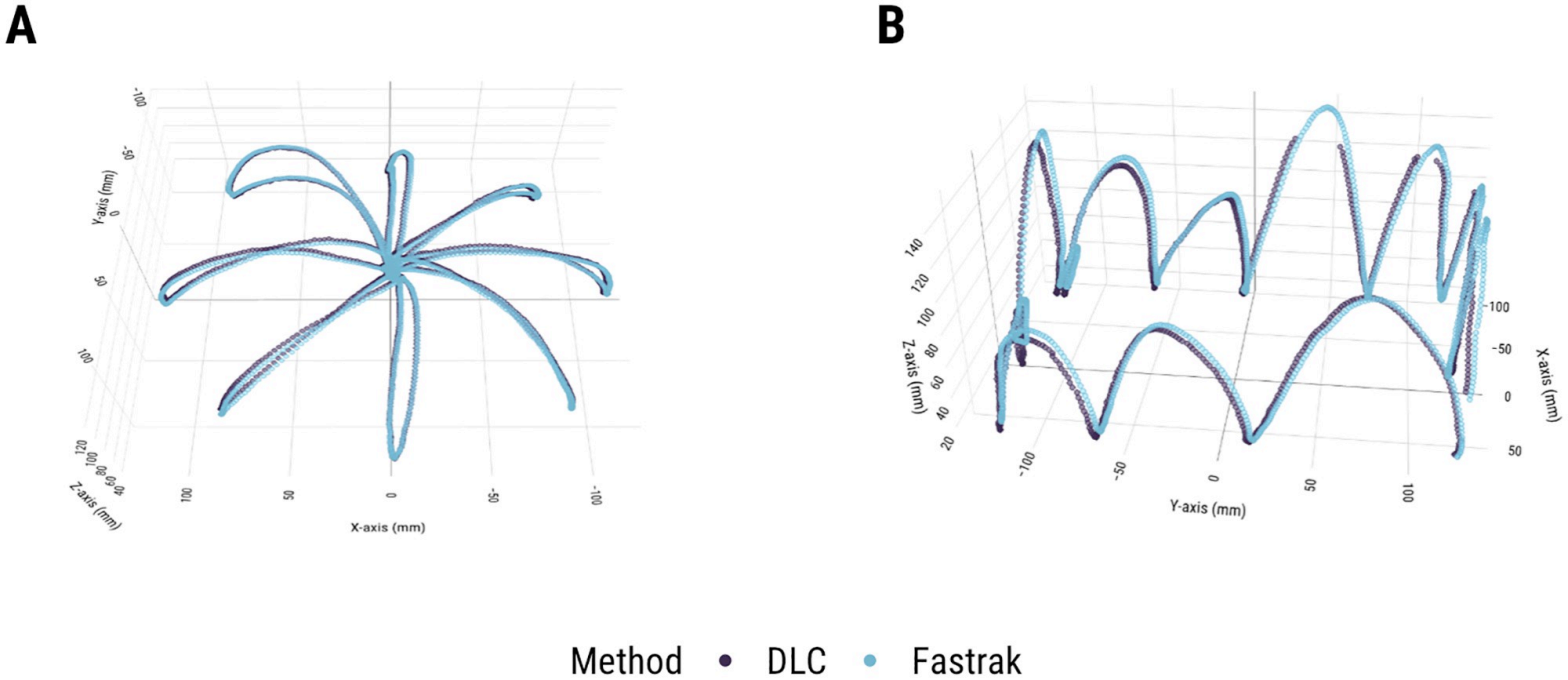
(a) Representative example of raw single trial data from the center-out (tool) task; (b) Representative example of raw single trial data from the zigzag (tool) task.

The task set for the validation consisted of two generic, representative upper limb motor tasks—a standard center-out reaching task and a more freeform, zigzag movement task, which was specifically chosen to evaluate DLC tracking performance with naturalistic 3D human arm motion. To ensure the validation results were not effector specific, we ran two versions of the task set–one performed with the index finger and another with a pointing tool (two thin conjoined wooden dowels). For each task, all trials were completed by one researcher. In the center-out task, the researcher made reaches from a central starting position to one of 8 targets arrayed around a circle (~12cm radius) with 45° spacing (Fig 2a). In the zigzag task, the researcher made movements around the visible edge of the testing area in a vertically oriented zigzag motion (Fig 2b). 20 trials were performed for each version of the 2 tasks (20 seconds of data recorded per trial in the center-out task and 10 seconds per trial in the zigzag task).

### Statistical analysis

To evaluate DLC tracking performance with naturalistic 3D human arm motion, participants performed two generic upper limb motor tasks—a standard center-out reaching task and a more freeform, zigzag movement task. The experimental tasks used can be considered to vary along two dimensions: type of task (centre-out, zigzag), and sensor location (tool, finger). After applying exclusion criteria described above, we had 47980 datapoints for the center-out tool task, 47649 for the center-out finger task, 23406 for the zig-zag tool task, and 22704 for the zig-zag finger task. This was considered sufficient for all reported analyses.

Agreement between concurrent DLC and Fastrak data was determined by calculating bias and limits of agreement (LOA) using the Bland-Altman method [11, 12], root mean square error (RMSE), and conducting a time series analysis. Bias, LOAs and RMSE were calculated using R, and time-series analyses were conducted in MATLAB (2019b). The Bland-Altman method is a common approach to quantify the agreement between two systems of measurement [13], and RMSE has been used as a metric of agreement in several similar motion-tracking measurement agreement studies [14–16]. Bias and RMSE estimate the average difference in measurements between DLC and Fastrak, whereas limits of agreement specify the interval that 95% of measurement differences fall within. For the time series analysis, cross-spectral coherence values were used to determine whether the same temporal structure was present in the DLC and Fastrak recordings [17, 18]. Zero-lag cross-correlation values were also calculated to determine the degree of sequential covariance between the DLC and Fastrak recordings [19, 20].

We treated our data as single measurements and computed bias and limits of agreement as outlined in [11, 12]. Note that despite having multiple trials of the same task, the data cannot be considered repeated measures because the freehand motion used in this task does not follow the exact same trajectory on every trial. When calculating RMSE, we considered Fastrak data as ‘observed’ and DLC data as ‘predicted’. No pre-processing or data smoothing was applied to the data prior to analysis. Cross spectral coherence measures the relationship or correlation between two time-series with respect to frequency and results in a value ranging from 0 to 1, where 1 corresponds to perfect spectral coherence. Here we report the average coherence calculated at each signal’s peak frequency, with each signal’s frequency spectrum calculated using MATLABs Fast Fourier Transform (FFT) function. Cross correlation measures the covariance between two time-series signals across a specific range of temporal lags. Given that we were interested in the sequential dependence of the two signals we calculated the zero-lag correlations between the Fastrak and DLC time-series (note that the maximum cross correlation was always at a lag of 0 or 1, with the latter due to trivial delays in data synchronization). Like a standard correlation, cross-correlations values range between -1 and 1, with values close to 1 indicating a high degree of (positive) sequential covariance (i.e., signal similarity).

All data, code, diagnostics, and mean-difference plots are available online (https://osf.io/mdcqs/).

## Results

**Fig 3:**
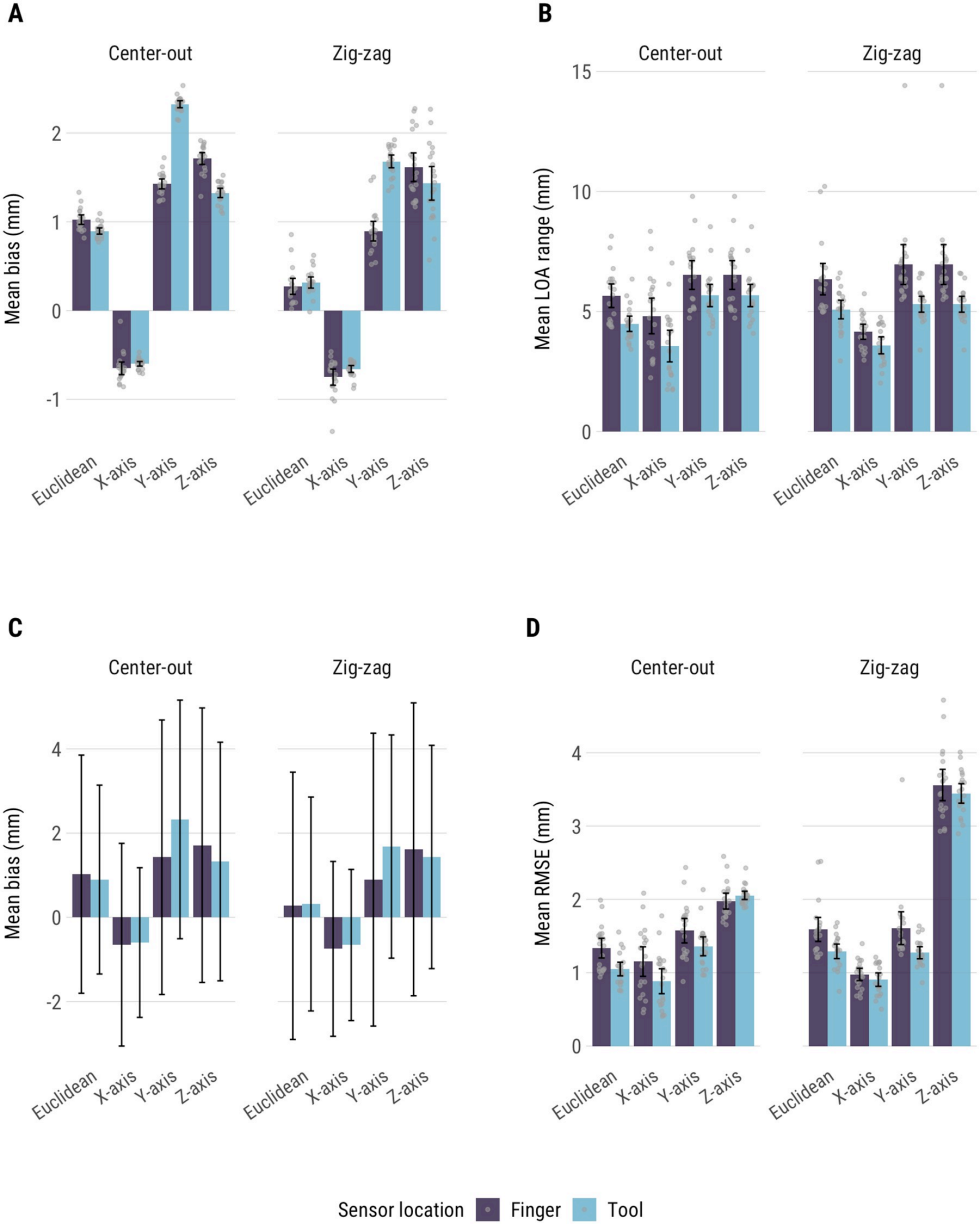
(A) Mean bias across the twenty trials, for each of the four tasks. Error bars are 95% confidence intervals and grey points are bias values for each trial (same for graphs C and D). (B) Ranges of limits of agreement across trials, for each of the four tasks. (C) Mean bias (bars) and limits of agreement (error bars) presented together. (D) Mean RMSE values across trials, for each of the four tasks.

We found extremely close agreement between DLC and Fastrak (summarized in Fig 3). Both bias estimates and RMSE indicate close agreement, with an average bias value of 0.8mm and an average RMSE of 1.4mm. The mean LOA range was 5.3mm, suggesting that most datapoints from DLC and Fastrak differ by no more than half a centimeter. Relative to Fastrak, DLC generally underestimated the distance from sensor position to reference point by 0.3–2.3mm (mean bias, Fig 3a). This was the case for all distance measures, except X-axis (left-right motion in camera view), where DLC overestimated the distance by 0.6–0.75mm. The overall pattern of bias was similar for center-out and zigzag tasks, but there were notable differences between finger and tool versions of the task. When the sensor was attached to the pointing tool, DLC showed more bias when estimating depth (Y-axis) than when the sensor was attached to the finger pad. It is not clear why DLC was more accurate when attached to the finger pad. The ranges of limits of agreement varied from 3.6mm to 7.0mm (i.e., from ±1.8mm to ±3.5mm relative to bias). Mean limits of agreement with respect to mean bias estimates are presented in Fig 3b. Limits of agreement vary more between trials than bias estimates (Fig 3a and 3c)–suggesting that it may be possible to get better agreement in principle, but not consistently so.

**Fig 4:**
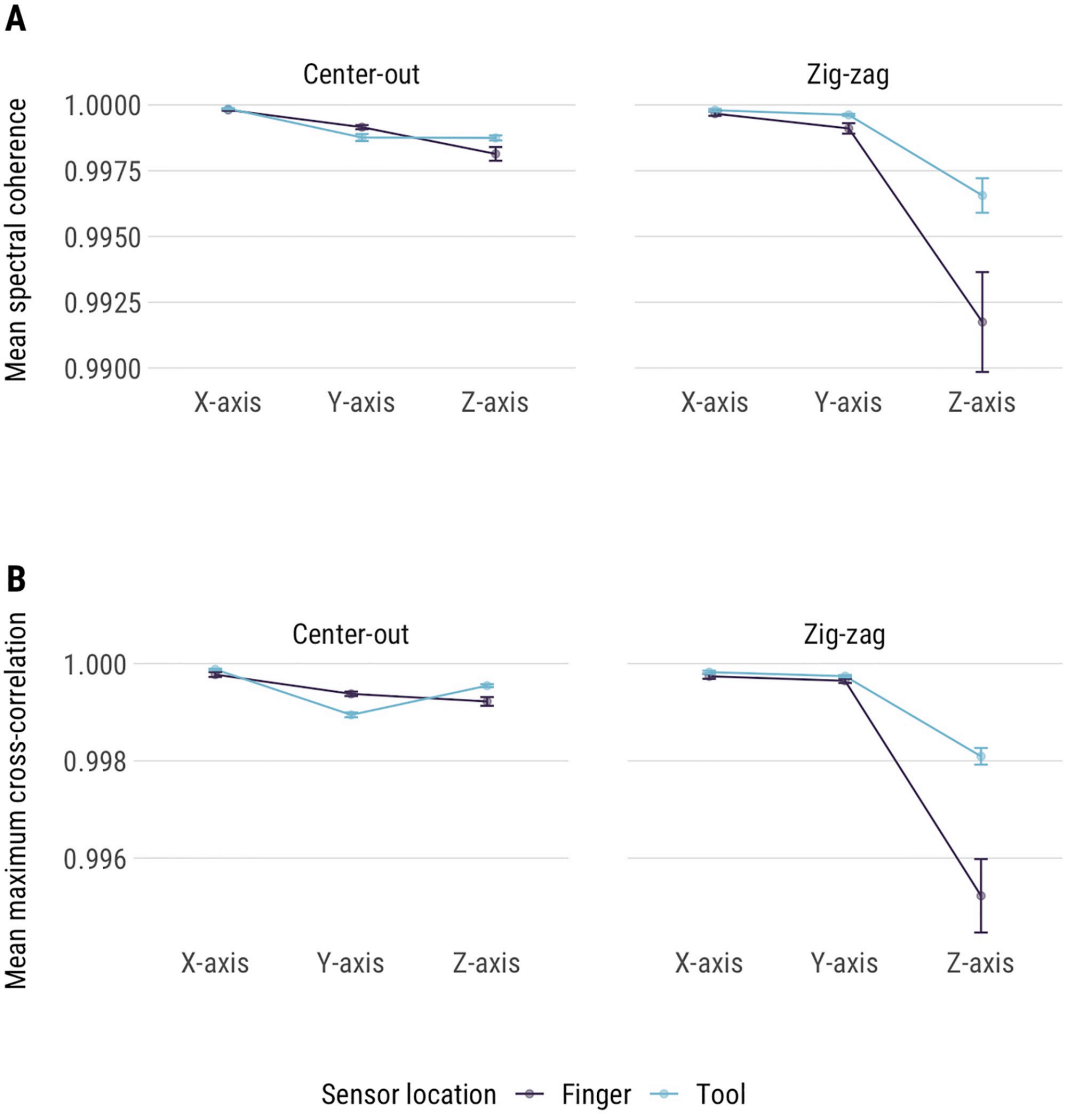
(A) Mean cross-spectral coherence values for all tasks. (B) Mean cross-correlation values for all four tasks. Error bars are 95% confidence intervals.

Both cross-spectral coherence and cross-correlation analysis revealed that there was excellent temporal agreement between the Fastrak and DLC recordings (Fig 4). Both indices have a maximum positive covariance value of 1, and the analysis resulting in values >0.99 for all three axes and across all tasks. As with other analyses, a minor reduction in temporal covariance was observed for Z-axis in the zigzag task, particularly for the finger version of the task. Overall, however, the results indicate that DLC preserves the temporal structure of human motion with the DLC and Fastrak recordings have very similar and synchronized motion patterns over time.

## Discussion

Measurement is a fundamental aspect of modern science [21]. When new measurement techniques or methods are adopted by a scientific field, careful validation is critical to ensure that these new methods are reliable and accurate. One commonly used approach to method validation–often called the gold standard or reference system approach–involves confirming that the new method agrees within acceptable limits with an established field-standard method [11, 12, 22].

Although deep learning-based approaches to markerless 3D pose estimation have taken neuroscience by storm, unfortunately, many of these tools remain unvalidated. Here, we report on the validation of one increasingly popular tool (DeepLabCut) against simultaneous measurements obtained from a reference measurement system (Fastrak) with well-known performance characteristics. We found that under tightly controlled experimental conditions, DLC can achieve extremely close (mm range) agreement with field-standard motion tracking systems such as Polhemus Fastrak, with 95% of the differences between Fastrak and DLC falling below 1 cm. The agreement was generally similar across the entire filming volume, as suggested by similar patterns in agreement measures in both center-out and zigzag tasks. Despite greater bias along the Y-axis in the tool version of both tasks, bias was similar between tool and finger versions along other dimensions. Limits of agreement were smaller for all tool task data. This suggests that overall, there was a small, yet slightly greater cost in accuracy when tracking more naturalistic motion. However, this cost appears to be well within acceptable levels for most tracking and task scenarios.

Whether the degree of agreement reported in this study is acceptable will depend on the specific needs of the researcher. We found the calibration step to be the least reliable element of the DLC pipeline and getting accurate measurements compared to ground truth may require several attempts and iterative adjustment of the camera calibration settings to get the desired result. Importantly, care must also be taken to synchronize the video recordings (see S1 File). If done well, however, excellent 3D kinematic data can be obtained using DLC with only a minor reduction in accuracy compared to electromechanical tracking systems like Fastrak. For many applications, these slight costs in accuracy will be small compared to those imposed by having to use wired sensors or contend with noisy data due to the presence of metal near, in, and around the workspace.

### Limitations

It is important to acknowledge some limitations of the current validation study, which constrain the conclusions one can draw from our work. Because our objective was to validate DLC against Fastrak, rather than test the conditions under which DLC performance breaks down, we actively sought to minimize as many sources of variation and noise in our datasets as possible. Given this objective, we constructed an experimental setup that enabled comparison of DLC and Fastrak performance when both were operating under near-optimal conditions. Consequently, we did not necessarily test or probe the full range of conditions in which DLC might be used by the research community. For example, video data was collected in tightly controlled laboratory conditions with uniform lighting, invariant camera angles and distances, using highly constrained task behaviors performed by a single subject to limit movement variability. These design choices were intentionally made to reduce noise in the data selection step of the DLC workflow, as DLC performance can be highly sensitive to the representativeness of the frames used in the training dataset [7]. Although these artificial conditions were important for validation purposes, they very likely depart from many real-world use cases in which luminance conditions, backgrounds, camera angles, and other factors will almost certainly vary across behavioral trials and sessions. Consequently, some caution must be taken when extrapolating DLC performance (or Fastrak performance, for that matter) to less precisely controlled, real-world contexts.

To obtain concurrent data, we used DLC to track the position of a Fastrak microsensor. For the tool-based versions of the tasks, crosshairs were drawn over the theoretical center of the embedded microsensor. In the reaching task, the microsensor was attached at the absolute center of the fingernail of the right index finger. These steps were intentionally taken to improve the visibility of the microsensor throughout the experiment, and consequently improve the accuracy and precision of the DLC labelling process. Like all deep learning network-based approaches, DLC performance depends on good input data, which heavily relies on consistent labelling of similar spots (e.g., the same joint or body part) across frames. Consistent labelling both within and across individuals can be extremely difficult in practice for many real-world applications of DLC, where readily identifiable visual features (i.e., markers) are not present (See S1 File for further discussion of the role of human error in DLC labelling). Consequently, this must be factored in when trying to extrapolate DLC performance observed in our study to other contexts in which labelling noise or variability is more likely to be in play.

## Conclusions

In this study, we validated one increasingly popular deep learning-based tool for markerless 3D pose estimation (DeepLabCut) against simultaneous measurements obtained from a reference system (Fastrak) with well-known performance characteristics. Our results confirm close (mm range) agreement between the two, indicating that under specific circumstances deep learning-based approaches can match more traditional motion tracking methods. Although more work needs to be done to determine the full range of performance characteristics and limitations of these new tools, this study should help build confidence within the research community when considering their use.

## Supporting information

S1 File (DOCX)
