# Supplementary material for: Validation of deep learning-based markerless 3D pose estimation
Source: PLoS One. 2022 Oct 20;17(10):e0276258. doi: 10.1371/journal.pone.0276258 (PMC9584509; doi:10.1371/journal.pone.0276258)
Supplement: S1 File — (DOCX) [file pone.0276258.s001.docx]

**S1 File**

The main aim of the study was to evaluate agreement between DLC and Fastrak. However, the process of obtaining 3D data from DLC involves multiple steps, each potentially introducing errors and noise. To investigate this, we ran a series of additional tests designed to reveal the influence of likely sources of noise in our DLC data: human error in labelling, network noise, camera lens distortions, and calibration inaccuracies.

*2D static test.* This test was designed to evaluate potential distortions coming from the camera lens, noise coming from different people labelling the frames, and random differences in the training process. We filmed a precisely manufactured ChArUco board [1] with known dimensions (Fig S1a), positioned ~55cm away and directly facing the camera. We filmed 20 trials, 1 second each, at 120fps. All recordings were merged into a single video file for easier processing. Three different people (DK, LL, VK) independently labelled a random selection of frames. The corners of five squares on the board (four approximately in the corners, one in the center, see Fig S1b) were labelled, with 20 labelled points in total.

We ran the network training process three separate times on each set of labelled frames, obtaining nine datasets. For each dataset, we calculated the Euclidean length of each square side in pixels, based on X and Y coordinates provided by DLC. Since the ChArUco board was directly facing the camera and labelling was performed on an object with known dimensions including a known fixed side length of each individual square (10mm), we can reasonably expect a fixed true value of the square side length in pixels as well.


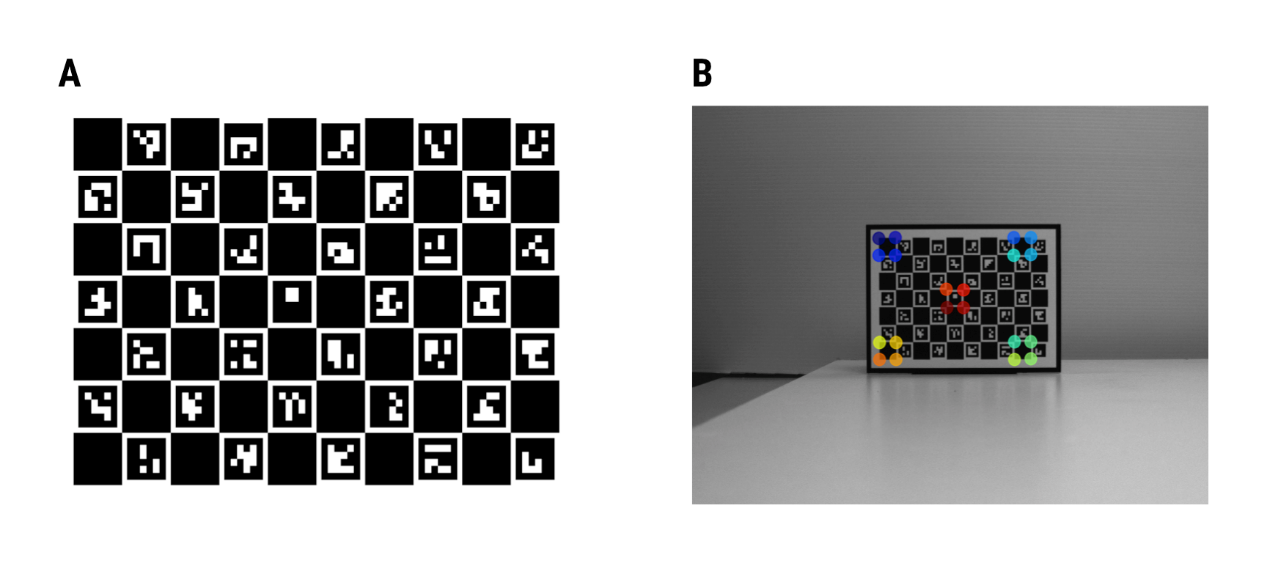


**Figure S1: (A) Pattern printed on the ChAruCo board used for 2D and 3D static tasks. (B) An example labelled frame from 2D static task, showing the points labelled for both 2D and 3D static tests.**

*3D static test.* This test was designed to verify that the process of translating 2D data into 3D coordinates is reliable and agrees with ground truth throughout the filming volume used in the main experimental tasks. We filmed a stationary ChArUco board (same as above) positioned in 6 different locations in the filming volume: at the front, middle, and back of the field of view, on either the left or the right half of it. The filming volume and the cameras were set up identically to the 3D dynamic tasks, and we filmed 20 trials of 1 second each. We labelled the same 20 points as in the 2D static test. The calibration video was filmed during the same session as the main experiment, and the calibration process was the same. After getting 3D coordinates for this dataset, we calculated Euclidian lengths of square sides in millimeters.

*Results*. For each static test, we calculated the mean and standard error (SE) of the square side lengths, for the relevant unit of analyses. For the 2D static test, we compared mean lengths between labelers and network training repeats. The differences between labelers were less than one pixel (see Table S1 and Figure S2a), suggesting that inter-labeler variability is unlikely to be a major determinant of DLC performance

**Table S1: Mean (SE) of square side lengths, in pixels, from 2D-static test.**

| Network training repeat | Labeller | | |
| --- | --- | --- | --- |
|  | DK | LL | VK |
| 1 | 22.78 (0.003) | 23.64 (0.006) | 23.34 (0.003) |
| 2 | 22.79 (0.004) | 23.44 (0.010) | 23.56 (0.003) |
| 3 | 22.78 (0.004) | 23.62 (0.009) | 23.43 (0.003) |

The differences between network repeats were negligibly small. For the 3D static test, the worst distortion occurred at the back left of the filming volume. But even there, DLC underestimates length by less than half a millimeter. DLC and the calibration process also appear to be highly reliable, as standard errors are extremely small (Table S2 and Figure S2b). This indicates that random differences in network training across sessions or experiments are unlikely to play a major role in DLC performance.


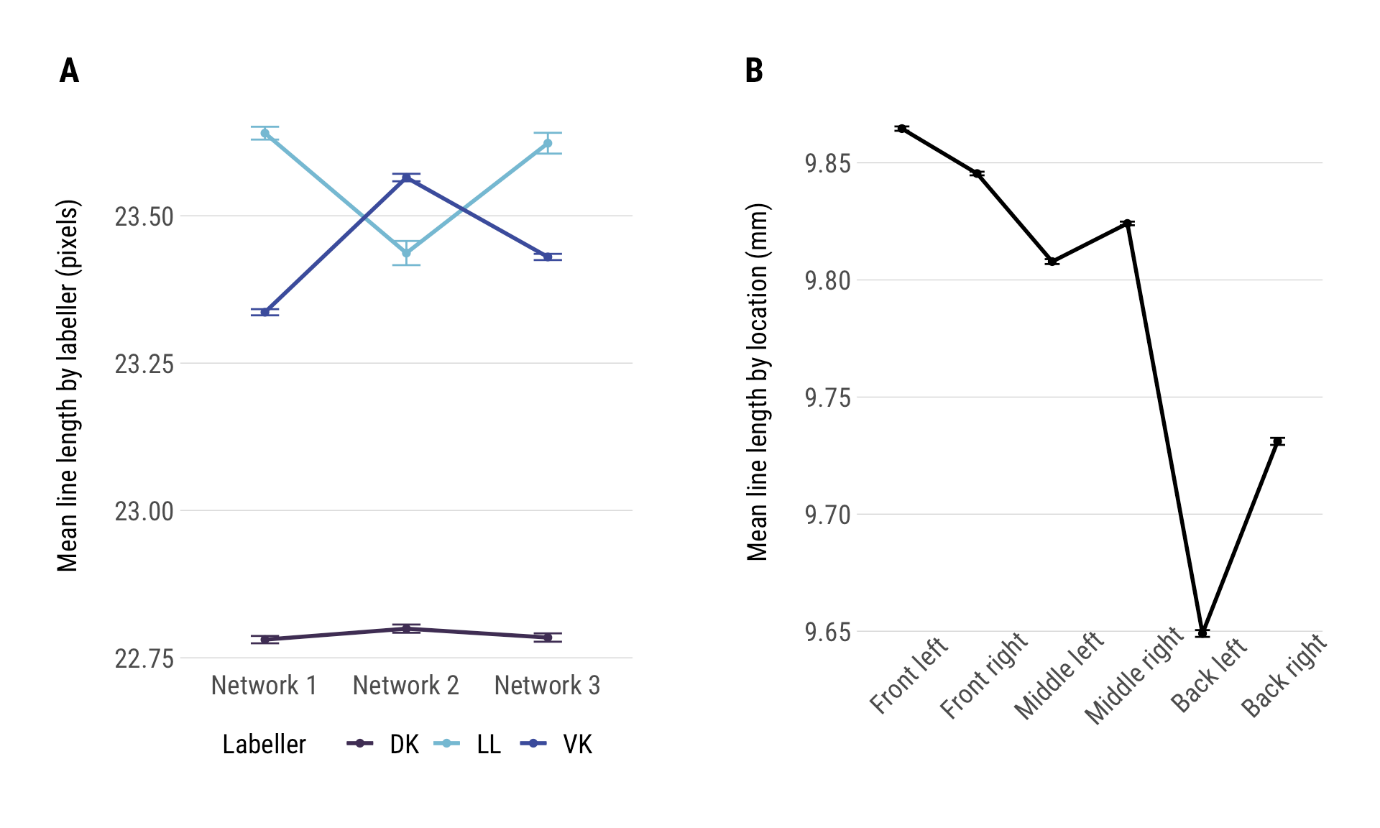


**Figure S2: (A) Mean line length (in pixels) for each labeler and network training repeat in the 2D static test. (B) Mean line length (in millimeters) in each of the six locations within the filming volume in the 3D static test. Error bars are 95% confidence intervals.**

**Table S2: Mean (SE) of square side lengths (in millimeters) at different filming locations in 3D-static test.**

| Position in filming space | Left | Right |
| --- | --- | --- |
| Front | 9.86 (0.0005) | 9.85 (0.0004) |
| Middle | 9.81 (0.0005) | 9.82 (0.0004) |
| Back | 9.65 (0.0007) | 9.73 (0.0008) |

Reference

1. Garrido-Jurado, S., Muñoz-Salinas, R., Madrid-Cuevas, F. J., & Marín-Jiménez, M. J. (2014). Automatic generation and detection of highly reliable fiducial markers under occlusion. *Pattern Recognition*, 47:2280-2292.
